# The Association between Neutrophil-to-Lymphocyte Ratio and Diabetic Depression in U.S. Adults with Diabetes: Findings from the 2009-2016 National Health and Nutrition Examination Survey (NHANES)

**DOI:** 10.1155/2020/8297628

**Published:** 2020-10-12

**Authors:** Jie Wang, Depu Zhou, Xiaokun Li

**Affiliations:** Department of endocrinology, Affiliated Hospital of Yanbian University, Yanji, Jilin, China

## Abstract

**Objective:**

To determine the association between neutrophil-to-lymphocyte ratio (NLR) and clinically relevant depressive symptoms in people with diabetes.

**Methods:**

This cross-sectional study was conducted among adults (age >18) with diabetes in the National Health and Nutrition Examination Survey (NHANES) between 2009 and 2016. NLR was calculated from complete blood count. Nine-item Patient Health Questionnaire (PHQ-9) was used to measure depression, with scores ≥10 indicating the presence of clinically relevant symptoms. Multivariable logistic regression was used to calculate the odds ratio (OR) with 95% confidence interval (CI) of clinically relevant depressive symptoms in relation to the NLR. We performed the smooth curve fitting and established a weighted generalized additive model to identify the nonlinearity of NLR and depression in diabetes patients. To account for the nonlinear relationship between NLR and depression in diabetes patients, weighted two-piecewise linear model was applied.

**Results:**

We included 2,820 eligible participants, of which 371 (12.4%) had clinically relevant depressive symptoms. In the unadjusted model, the OR (95% CI) of clinically relevant depressive symptoms for the second (NLR 1.75-2.57) and third (NLR >2.57) were 1.24 (0.90, 1.70) and 1.68 (1.23, 2.30), respectively, compared to the reference group (NLR < 1.75). After controlling for potential confounding factors, NLR was significantly associated with clinically relevant symptoms (odds ratio = 1.57, 95% confidence interval: 1.13–1.87; *P* for trend = .0078). Nonlinear relationships were observed, and a two-piecewise linear regression model was established. The inflection point of NLR was 2.87. To the left of the inflection point (NLR ≤ 2.87), the OR (95% CIs) was 1.33 (1.07–1.66) (*P* < .031).

**Conclusions:**

Elevated levels of NLR are independently associated with increased odds of clinically relevant depressive symptoms in people with diabetes. Prospective study is needed to further analyze the role of NLR in depression in diabetic patients.

## 1. Introduction

The prevalence of diabetes mellitus (DM) has been rapidly increasing, posing enormous burden for individuals, families, and countries [1, 2]. Depression is highly prevalent in diabetic patients [3–5], and a depressive disorder is nearly twice as prevalent in individuals with DM as in the general population [6]. Compared with people suffering from diabetes but without depression, those who suffer from both diseases also tend to be less adherent to diabetes therapy, spending higher costs and having a higher risk of death [7]. For these reasons, it is urgent to identify early prediction and preventive measures to relieve the burden in diabetic patients with depression.

Low-grade inflammation plays an important role in the mechanism of depression [8]. Studies have clarified that antidepression medicine can temporally ameliorate depression symptoms via decreasing the expressions of proinflammatory factors while increasing the levels of anti-inflammatory factors. In animal model, high-fat diet activates increase the release of inflammatory cytokines, leading to depression [9], and the activation of Nod-like receptor pyrin domain3 (NLRP3) inflammasomes to produce interleukin-1 (IL-1) results in the depressive-like behavior and insulin resistance [10].

Neutrophil-to-lymphocyte ratio (NLR) is a widely accepted biomarker for the evaluation of overall inflammation status [11, 12]. It is cost-effective, an easily available index of the inflammatory levels that is easily obtained from complete blood counts, based on neutrophil and lymphocyte counts [12]. Several studies revealed that increased NLR is associated with psychiatric disorders, especially depression [13–16]. Most of the past research shared the same limitation of rather small sample sizes. Additionally, some important confounding aspects were out of consideration in the research, including body mass index (BMI), annual household income, and cardiovascular history. In diabetic patients with chronic inflammation, the NLR level might be a predictive and preventive biomarker associated to depression, which has never been studied.

However, there has no study to investigate the association between NLR and clinically relevant depressive symptoms in people with diabetes. The present study was conducted to explore the relationship between clinically relevant depressive symptoms and NLR levels in diabetic patient with a great range of potential confounders adjusted.

## 2. Methods

### 2.1. Study Population

The study followed the Strengthening the Reporting of Observational Studies in Epidemiology (STROBE) statement [17, 18]. This cross-sectional study applied existing information in data files for public use from the National Health and Nutrition Examination Survey (NHANES) from 2009 to 2016 [19]. Data used in this study are publicly available (https://www.cdc.gov/nchs/nhanes/). The Centers for Disease Control and Prevention (CDC) initiated NHANES, a strict, long-term, and large-scale study of the general population. Through interviewing, collection of blood samples, self-report questionnaires, and measurement of anthropometry, NHANES aimed to obtain information regarding vital and health statistics among the general American population. The evaluation was performed at mobile examination centers (MECs), and the measures included physical examinations and household interviews. For our analyses, participants were selected in four cycles of NHANES survey (2009–2010, 2011–2012, 2013–2014, and 2015–2016) to assess the association between NLR and diabetic depression.

**Figure 1:**
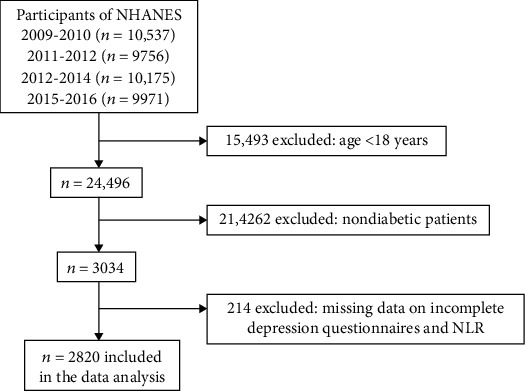
Case selection schematic for exclusion or inclusion in the studied sample.

For this study, a total of 40,439 individuals of all ages took part in the NHANES from 2009 to 2016, and our analyses were limited to 24,496 people older than 18 years of age. We excluded participants who had missing data on incomplete depression questionnaires, DM questionnaire, and NLR. Ultimately, 2,820 participants were included in the analysis of this study (Figure 1).

### 2.2. Assessment of Depression Symptoms

The Patient Health Questionnaire (PHQ-9) is a screen for depression containing nine items regarding the frequency of depression symptoms [20–22]. PHQ-9 was applied face-to-face during the MEC interview to evaluate depression symptoms over the previous two weeks. Each item is scored on a scale from 0 to 3, and scores in total lay between 0 and 27. The symptoms were as follows: (a) psychomotor disturbances, (b) low self-esteem, (c) appetite changes, (d) anhedonia, (e) sleep disturbance, (f) concentration problems, (g) depressed mood, (h) suicidal ideation, and (i) fatigue.

Depression scores were classified as four groups as follows: “severe” (20–27), “moderately severe” (15–19), “mild” (5–9), “moderate” (10–14), and “none or minimum” (0–4) [23]. We dichotomized these data to distinguish between participants who scored 9 or less and those who scored ≥10 or more; the latter suggested clinically relevant depression [24].

### 2.3. Assessment of NLR

Lymphocyte and neutrophil counts were evaluated using automated hematology analyzing devices and were expressed as ×1,000 cells/mm^3^. NLR was measured as neutrophil count-to-lymphocyte count ratio. To determine the exact relationship between the parameters in hematology and depression symptoms, we treated these as continuous variables in tertiles to apply the available data more efficiently and flexibly.

### 2.4. Study Variables

Age, sex (male and female), and annual household income were included as characteristics of self-reported social demography. According to races, respondents were categorized into five groups (Mexican American, Non-Hispanic Black, Non-Hispanic White, other Hispanic, and other) from 2009 to 2010 and six groups (we added an extra group of Non-Hispanic Asian) from 2011 to 2016. According to level of education, respondents were classified into groups as follows: for subjects aged more than 20 years–graduation from college or above, some degree from college or associate degree, graduation from high school, General Equivalent Diploma (GED), 9^th^–12^th^ grade but with no diploma, or less than 9^th^ grade; for those who aged 18–19 years–more than high school, GED, high school graduate, 1^st^–12^th^ grade with no diploma, and never attended schools or kindergarten only. According to standard procedures, height and weight were recorded when physical examinations were performed in the subjects' home or in a MEC. BMI was calculated as weight in kilograms divided by the square of height in meters [25].

Lifestyle features consisted of smoking status and alcohol drinking. According to smoking status, participants were grouped as current smokers (no less than 100 cigarettes during the whole life and still smoking now), never smokers (less than 100 cigarettes without smoking now), and former smokers (no less than 100 cigarettes during the whole life and quitting now).

We also obtained information about chronic diseases that were suspected to correlate with depression, including diabetic retinopathy (DR), heart failure (HF), stroke, and coronary heart disease (CHD). We defined DM as DM history or subjects taking antidiabetic medicines or insulin now.

### 2.5. Statistical Analyses

To investigate the relationship between NLR and depression in diabetes patients, the statistical analysis process was divided into three steps. First, the study participants were subdivided into three groups according to the NLR (tertiles). Continuous data were expressed as mean ± standard deviation (SD), and categorical variables were expressed as frequency or percentage. Differences in baseline characteristics between tertiles of NLR were compared via a Kruskal–Wallis H test in continuous variables and *χ*^2^ tests in categoric variables. Second, logistic regression analysis was performed to determine the relationship between NLR and clinically relevant depression symptoms in diabetes patients. In model 1, no covariates were adjusted; in model 2, only age, sex, and race were adjusted; model 3: adjusted confounders were age, sex, race, education, household annual income, smoking status, alcohol consumption, and BMI (normal weight, overweight, obese); and model 3: adjusted confounders were model 3 plus adjusted for HbA1c in quartiles, chronic conditions including HF, stroke, DR and CHD, glucose-lowering drugs, and insulin use. We conducted subgroup analysis to increase comparability between two groups with these being: age and sex. Thirdly, to identify the nonlinearity of NLR and depression in diabetes patients, we performed the smooth curve fitting (with the method of penalized spline) and established a weighted generalized additive model. Due to limitations of classification analysis, the point of inflection was primarily figured out by applying a recursive algorithm. Afterwards, on the two sides of the point of inflection, a model of weighted two-piecewise linear regression was established. According to the *P* values in log likelihood ratio test, we found the model that fitted the best (a two-piecewise linear regression model compared with the linear regression model).

All analyses were conducted using the R statistical software (Version 3.6.2, http://www.r-project.org). All *P* values were two-sided, and *P* values < .05 were considered statistically significant.

## 3. Results

### 3.1. Baseline Characteristics of Selected Participants

A total of 2,820 eligible participants aged ≥18 years had data on NLR and depressive symptoms. The participants included 1,448 females and 1,372 males with a mean age of 61.5 ± 15.0 years, and mean NLR of 2.4 ± 1.6. The characteristics in relation to NLR are summarized in Table 1. Among the samples we analyzed, the participants were divided into tertiles according to the NLR < 1.75 (933); 1.75–2.57 (946), and >2.57 (941). Compared with NLR < 1.75 (1.75–2.57), participants with higher NLR (>2.57) were more likely to be elderly, female, obese, and never smokers; there were a greater number of chronic conditions, including DR, CHD, and stroke; they also had lower household incomes and lower levels of education.

### 3.2. Association between NLR and Clinically Relevant Depressive Symptoms in Diabetics

We constructed three models to assess the independent effects of NLR and clinically relevant depressive symptoms in diabetics after adjusting for other potential confounders.  Table 2 displays effect sizes (ORs) and 95% CIs.

In our unadjusted model, the OR (95% CIs) of clinically relevant depressive symptoms for the second (NLR 1.75–2.57) and third (NLR > 2.57) tertiles were 1.24 (0.90, 1.70) and 1.68 (1.23, 2.30), respectively, compared to the reference group (NLR < 1.75). This association remained significant after adjustment for age, sex, race, education, household annual income, smoking status, BMI, HbA1c in quartiles, and chronic conditions including HF, stroke, DR, and CHD, and medication use, including glucose-lowering drugs and insulin use (OR = 1.57, 95% CI: 1.13–1.87; Table 2). This trend was statistically significant (*P* = .0078). At the NLR quartile, a similar relationship has also been observed.

Subgroup analysis showed the associations between the NLR level and depression of DM patients with different parameters (Table 3). High NLR levels are independently related to clinically relevant depressive symptoms in age <65 [OR (95% CI), 1.86 (1.32, 2.61)], but not 65 [OR (95% CI), 1.09 (0.69, 1.72)].

### 3.3. Dose-Response Relationship between NLR and the Risk of Clinically Relevant Depressive Symptoms in Diabetics

**Figure 2:**
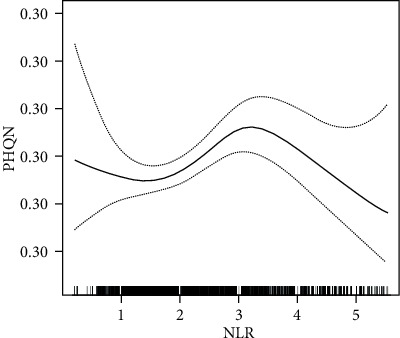
Relationship between NLR and logit transformed clinically relevant depressive symptoms.

To demonstrate nonlinearity of NLR and clinically relevant depressive symptoms in diabetics, we performed smooth curve fitting (using the penalized spline method) (Figure 2). After adjusting for covariates, nonlinear relationships were observed. Because of the limitations of classification analysis, a two-piecewise linear regression model was established, and the inflection point of NLR was 2.87 (Table 4). To the left of the inflection point (NLR ≤ 2.87), the OR (95% CIs) was 1.33 (1.07–1.66) (*P* < .031). When NLR was greater than 2.87, the relationship between NLR and clinically relevant depressive symptoms was not detected [OR = 0.99, 95% CI (0.87, 1.12)].

## 4. Discussion

To the best of our knowledge, our study is the first case-control study to determine the relationship between NLR and clinically relevant depressive symptoms in people with diabetes. We found that diabetic patients suffering from depression demonstrated a significantly higher level of NLR than did those without depression. Elevated NLR was significantly related to an elevated risk of depressive conditions in diabetic patients. Following the control of possible confounding factors, whether men and women, the association remained.

NLR level is helpful in demonstrating inflammatory activation in psychiatric disorders and could work as a reproducible biomarker in systemic inflammatory activities that could be detected routinely [11, 26]. In the general population, Meng et al. reported that the elevated NLR level was independently associated with depression symptoms among females, but not among the males [27]. Demircan et al. reported that patients suffering from depression disorders had significantly higher NLR than control patients [15]. Demir et al. reported that NLR was greater in 41 patients with depression than in a control group [28]. However, these studies had small sample sizes, limiting the generalization of their findings. Diabetes naturally causes increased NLR, and the association between diabetic depression and NLR has never been studied. In the present study, we found that NLR was an independent risk factor for depression in a large sample of people with diabetes. We can use the NLR, which is a simple and effective marker of inflammation and immunity, to assess patient's status.

Inflammatory cytokines are essential biomarkers for treatment assessment, diagnosis, and prognosis of depression [29, 30]. However, high costs and difficulties in availability are barriers to routine measurement of inflammatory cytokine expression patients suffering from depressive symptoms. WBC are relatively inexpensive and are available [31]. Subtypes of WBC including monocytes, neutrophils, and lymphocytes indicate several aspects of inflammation activities in various chronic diseases [31]. The other types of neutrophils are important for initiating and modulating both adaptive and innate immunity processes. In the context of inflammation processes, neutrophils are the first responding immune cells and modulate immune cell recruitment at inflammation sites [32]. Importantly, neutrophil activation may induce oxidative stress via releasing reactive oxygen species (ROS) [33], and ROS may participate in the pathogenesis of depression [34]. Lymphocytes are important components of circulating leukocytes, mediating adaptive immunity, and closely collaborating in innate immunity processes [35]. The NLR, which combined the neutrophil counts and the lymphocyte counts into a more comprehensively biomarker, may have an evaluation value of overall inflammation status in diabetic depression patients.

Compared with previous studies, the strengths of our study include a large sample size. We also adjusted for a considerable number of potential confounding factors that can influence the association between inflammatory markers and depression. Inevitably, our research has some limitations. First, because of the essence of cross-sectional research, we could only provide weak evidence for the association between depression and NLR, and it is difficult to draw causal inferences. Therefore, designs of prospective studies are more suitable to solve this problem. Second, data were collected from only one routine blood test. Neutrophils have shorter life spans, and they rapidly turn over. For these reasons, serial neutrophil counts may be much more helpful than single measurement during an admission. Taken together, these findings suggest that future studies are required to validate the results of this study. NLR should be studied in more prospective cohort research involving depression.

## 5. Conclusion

In summary, we provided the first evidence that elevated NLR is independently associated with increased odds of clinically relevant depressive symptoms in people with diabetes. This finding needs to be confirmed in well-designed cohort studies.

## Figures and Tables

**Table 1:** Characteristics of the study patients according to NLR^a^.

Characteristics	<1.75	1.75-2.57	>2.57	*P* value
*N*	933	946	941	
Age (years)	58.7 ± 15.2	61.2 ± 14.3	64.0 ± 14.5	<.001
Sex (male, %)	57.2	47.0	41.8	<.001
Race (%)				<.001
Mexican American	19.5	19.3	16.4	
Other Hispanic	15.2	14.3	14.6	
Non-Hispanic white	23.5	27.7	27.8	
Non-Hispanic black	27.2	24.9	26.8	
Other	14.6	13.7	14.5	
Household income (%)				<.001
≤$20000	11.5	20.7	15.2	
$20000–74999	34.4	40.1	42.6	
≥$75000	54.6	39.2	37.3	
Education (%)				<.001
Less than high school	11.0	15.5	20.6	
High school	34.4	38.7	37.3	<.001
Above	54.7	45.8	42.1	
Marital status				.061
Married/living with partner	66.3	71.0	70.6	
Widowed/divorced/separated	17.6	17.1	16.8	
Never married	16.1	11.8	12.5	
Smoking (%)				<.001
Never smoker	55.6	59.5	64.3	
Former smoker	25.3	26.5	22.7	
Current smoker	19.1	14.0	13.0	
Alcohol drinking (yes, %)	36.1	35.9	36.5	<.041
BMI (kg/m^2^)	32.0 ± 7.2	32.5 ± 7.5	32.8 ± 8.1	.258
SBP (mmHg)	131.4 ± 19.4	131.4 ± 20.0	132.0 ± 20.3	.786
DBP (mmHg)	69.5 ± 14.1	68.3 ± 14.2	66.7 ± 14.2	<.001
DM-related characteristics				
Fasting glucose(mg/dL)	145.8 ± 54.3	155.4 ± 60.6	159.6 ± 60.6	<.001
HbA1c (mg/dL)	7.0 ± 1.7	7.3 ± 1.8	7.5 ± 1.8	.001
Taking insulin	19.5	22.6	26.9	
DR (yes, %)	23.5	27.7	28.9	.020
HF (yes, %)	10.9	9.1	10.1	.701
CHD (yes, %)	7.9	11.1	13.9	<.001
Stroke (yes, %)	6.8	9.3	11.4	.003
Depressive symptoms (yes, %)^b^	12.6	13.5	17.1	.022

^a^All estimates are weighted to be nationally representative. ^b^Depressive symptoms measured using Patient Health Questionnaire (PHQ-9) and depressive symptoms (PHQ-9≥10). Abbreviations: BMI: body mass index; SBP: systolic blood pressure; DBP: diastolic blood pressure; CHD: coronary heart disease; HF: heart failure; DR: diabetic retinopathy; and DM: diabetes mellitus.

**Table 2:** Associations of NLR with clinically relevant depressive symptoms among adults in NHANES (2009–2016).

NLR	Unadjusted	Multivariable-adjusted^a^	Multivariable-adjusted^b^	Multivariable-adjusted^c^
	OR (95% CIs)	OR (95% CIs)	OR (95% CIs)	OR (95% CIs)
Tertile				
<1.75	Ref.	Ref.	Ref.	Ref.
1.75-2.57	1.24 (0.90, 1.70)	1.20 (0.90, 1.60)	1.16 (0.86, 1.58)	1.08 (0.82, 1.43)
>2.57	1.68 (1.23, 2.30)	1.78 (1.34, 2.35)	1.74 (1.31, 2.32)	1.57 (1.13, 1.87)
*P* for trend	0.0009	<0.0001	0.0023	0.0078
Quartiles				
<1.56	Ref.	Ref.	Ref.	Ref.
1.56-2.13	1.08 (0.77, 1.51)	1.07 (0.74, 1.55)	1.07 (0.76, 1.50)	1.25 (0.85, 1.83)
2.13-2.86	1.41 (1.02, 1.96)	1.44 (1.01, 2.05)	1.40 (0.99, 1.95)	1.46 (0.99, 2.14)
>2.86	1.85 (1.34, 2.56)	1.71 (1.19, 2.46)	1.79 (1.29, 2.50)	1.62 (1.10, 2.39)
*P* for trend^d^	<0.0001	0.0010	0.0001	0.0134

Abbreviation: NLR: neutrophil-to-lymphocyte ratio; NHANES: National Health and Nutrition Examination Survey; Ref.: reference; OR: odds ratio; CI: confidence interval. ^a^Adjusted for age, sex, and race. ^b^Adjusted for age, sex, race, education, household annual income, smoking status, alcohol consumption, and BMI (normal weight, overweight, obese). ^c^Adjusted for all covariables in b plus adjusted for HbA1c in quartiles, chronic conditions including HF, stroke, DR and CHD, glucose-lowering drugs, and insulin use.

**Table 3:** Subgroup analysis of the associations between neutrophil–lymphocyte ratio with depressive symptoms.

Subgroups	NLR		
	<1.75	1.75-2.57	>2.57
Age (years)			
<65	Ref.	1.26 (0.89, 1.77)	1.86 (1.32, 2.61)
≥65	Ref.	0.85 (0.52, 1.40)	1.09 (0.69, 1.72)
Sex			
Male	Ref.	1.05 (0.55, 2.06)	1.34 (0.74, 2.42)
Female	Ref.	1.15 (0.75, 1.27)	1.69 (1.13, 2.52)

Abbreviation: NLR: neutrophil-to-lymphocyte ratio; Ref: reference. ^a^Adjusted for all covariables in age, sex, race, education, household annual income, smoking status, alcohol consumption, BMI, HbA1c in quartiles, chronic conditions including HF, stroke, DR and CHD, glucose-lowering drugs, and insulin use.

**Table 4:** Threshold and saturation effect analysis of NLR on depressive symptoms.

NLR	OR (95% CIs)^a^
Standard logistic regression model	1.07 (0.99, 1.15)
Fitting model by two-piecewise linear regression	
Inflection point of NLR	2.87
≤2.87	1.33 (1.07, 1.66)
>2.87	0.99 (0.87, 1.12)
*P* for likelihood	0.031

Abbreviation: NLR: neutrophil-to-lymphocyte ratio: OR: odds ratio; CI: confidence interval. ^a^Adjusted for age, sex, race, education, household annual income, smoking status, alcohol consumption, BMI, HBA1C in quartiles, chronic conditions including HF, stroke, DR and CHD, and medication use including glucose-lowering drugs and insulin use.

## Data Availability

All the data used to support this study are available from the corresponding author upon request.
